# The Role of Nutraceutical Supplements, Monacolin K and Astaxanthin, and Diet in Blood Cholesterol Homeostasis in Patients with Myopathy

**DOI:** 10.3390/biom12081118

**Published:** 2022-08-14

**Authors:** Ines Villano, Marco La Marra, Salvatore Allocca, Ciro Rosario Ilardi, Rita Polito, Chiara Porro, Sergio Chieffi, Giovanni Messina, Vincenzo Monda, Girolamo Di Maio, Antonietta Messina

**Affiliations:** 1 Department of Experimental Medicine, Section of Human Physiology and Unit of Dietetic and Sport Medicine, University of Campania “Luigi Vanvitelli”, 80138 Naples, Italy; 2 Department of Psychology, University of Campania “Luigi Vanvitelli”, 81100 Caserta, Italy; 3 Department of Clinical and Experimental Medicine, University of Foggia, 71122 Foggia, Italy; 4 Department of Movement Sciences and Wellbeing, University of Naples “Parthenope”, 80138 Naples, Italy

**Keywords:** monacolin k, astaxanthin, antioxidant, cholesterol, diet, dietary supplement, dyslipidemia, myopathy

## Abstract

Several studies suggest that different combinations of nutraceutical supplements may improve the lipid profile, representing a viable alternative to statins. However, their effects on individuals with myopathy need to be investigated. The aim of our study was to explore the mid- and long-term physiological effects of monacolin k (5 mg) and astaxanthin (0.1 mg) supplements in association with a low-energy/fat diet in a group of subjects with mild myopathy. Eighty subjects (44 women) took part in this observational study. Participants were assigned to the experimental group (EG, *n* = 40, 24 women) treated with a low-energy/fat diet (1200–1500 Kcal/day and 15–20% lipids) in combination with monacolin k (5 mg) and astaxanthin (0.1 mg) supplementation, and to the control group (CG, *n* = 40, 20 women) treated only with a low-energy/fat diet (1200–1500 Kcal/day and 15–20% lipids). BMI and biochemical parameters (blood glucose, total cholesterol, HDL, LDL, triglycerides, C-reactive protein (CRP) and creatine phosphokinase-CPK) were collected at baseline (T0), after 12 (T1) and 24 (T2) weeks. A mixed factorial ANOVA was performed to determine if there were significant main effects and/or interactions between time and treatment. Treatment (EG vs. CG) was entered as the between-subjects factor and time (T0 vs. T1 vs. T2) as the within-subject factor. We found a significant improvement in total cholesterol, HDL, LDL, PCR and CPK parameters in EG compared with CG. Our results highlight the efficacy and safety of combined use of monacolin k (5 mg) and astaxanthin (0.1 mg) in combination with a low-energy/fat diet in the treatment of dyslipidemia.

## 1. Introduction

Dyslipidemia is a well-known risk factor for cardiovascular disease (CVD) [1,2]. A combination of several lipid fraction abnormalities, such as hypercholesterolemia and hypertriglyceridemia, is frequently observed, especially in obese individuals [3]. In particular, the CVD risk is directly related to prolonged exposure time of elevated plasma Low-Density Lipoprotein Cholesterol (LDL-C) [4]. Consequently, management of dyslipidemia and application of lipid-lowering therapies have been suggested as possible tools to prevent dyslipidemia-related outcomes, such as CVD [5].

Statins, which act as hydroxy-methyl-glutaryl-coenzyme A (HMG-CoA) reductase inhibitors, are lipid-lowering drugs that control plasma cholesterol concentration and keep them in the physiological range [6,7]. They are used for the treatment of hypercholesterolemia as they inhibit HMG-CoA reductase, a key enzyme involved in cholesterol biosynthesis [6,7].

Although their efficacy is well-established, their long-term use is controversial, mainly because of the possible adverse events that may arise when used for an extended period. Scientific literature highlights skeletal muscle implications, metabolic deficits, and neurological *sequelae* [7,8,9] among these possible adverse events. These side effects can be defined as “statin-associated symptoms” (SAS) and as myopathies, also called “statin-associated muscle symptoms” (SAMS) [6,7,10]. SAMS are the most common manifestation, and range from mild myalgia to rhabdomyolysis [10,11,12]. The clinical presentation of myopathy is heterogenous (e.g., muscle pain, weakness, cramps and increase in serum creatine phosphokinase—CPK) [7,11]. Therefore, while there are several parameters used to quantify SAMS in clinical practice (e.g., CPK elevations, muscle weakness), SAMS assessment depends mainly on clinician rating [7,13]. The mechanism of statin myotoxicity seems to be related to the indirect and direct effects of statins. Several conditions may be implied, such as decreased hepatic influx activity of statin membrane transporters; reduced activity of statin metabolizing enzymes; and reduced hepatic and muscular efflux membrane transporters activity [14]. Myocytes and hepatocytes express several influx transporters for statins which results in the consequent accumulation of statins in myocytes [14]. Here, statins may damage the sarcoplasm and/or mitochondrial functions with disruption of the mitochondrial respiratory chain and decreased ATP production, reactive oxygen species (ROS) increase, leak of cytochrome c, Ca^2+^, and CPK [14,15]. The coenzyme Q10 is a key component of mitochondrial biochemistry to produce ATP and its synthesis takes place via a series of specific reactions starting from mevalonic acid [16]. The HMG-CoA reductase inhibitors like statins inhibit mevalonic acid production with consequent coenzyme Q10 deficiency inducing mitochondrial dysfunction and SAMS [16]. Moreover, HMG-CoA reductase is also expressed in skeletal muscle where statins inhibit the mevalonate pathway in the myocyte more efficiently than in the hepatocyte, reducing the formation of the metabolites that are required for the posttranslational modification of essential regulatory proteins [17,18,19]. All of these factors contribute to the activation of apoptosis, proteolysis, and muscle remodeling and in turn, could be responsible for fatigue, cramps, myalgia, and elevation of serum CPK [15]. Furthermore, the risk of statins-associated side effects increases in multi-treated patients (polypharmacy) as a consequence of possible drug–drug interactions that lead to statin or statin-derived metabolite accumulation [6,20,21]. As a result of the above side effects, patients show a decreased adherence to statin-based therapy, with an increasing risk of emerging/worsening cardiovascular outcomes [7,22].

The research in this area is aimed at developing functional strategies to manage both the dyslipidemia and the statin-related symptoms [13,23,24]. However, alternative lipid-lowering therapies, such as nutraceuticals, appear to be a promising field of investigation. Red yeast rice (RYR) has been often used as an alternative therapy for dyslipidemic patients that are intolerant to statins [25,26]. RYR is obtained by the fermentation of rice (*Oryza sativa*) by the fungus *Monascus purpureus* producing several metabolites, including a red pigment and the monacolins [4]. Among the different monacolins, monacolin K (MK) is mainly responsible for cholesterol-lowering effects. It inhibits HMG-CoA reductase, enhancing lipid metabolism [4,25,26,27]. It has been shown that MK is associated with a reduction in plasma levels of LDL-C, total cholesterol, and high-sensitivity C-reactive protein (CRP) [4]. However, mixed results are available; indeed, it would appear that RYR also manifests statin-like adverse reactions, such as myopathies (myalgia and CRP elevation) [25,28,29]. Moreover, monacolin k associated to statins could reinforce statin-side effects, inducing rhabdomyolysis due to their pharmacokinetic and pharmacodynamic interactions [30].

Evidence suggests that nutraceutical combinations might be able to improve the lipid pathway, recruiting and combining several specific mechanisms [31,32]. Recent studies propose RYR in combination with other nutraceuticals such as berberine, policosanol, astaxanthin, and coenzyme Q10 to decrease lipid and glucose levels in dyslipidemic subjects [27,31,32]. Furthermore, combination with antioxidants has been linked to both decreased CRP and endothelial dysfunction [27,33,34,35,36,37].

Astaxanthin (AX), a natural carotenoid contained in seafood, has been shown to have hypolipidemic and antioxidant activity that protects biological membranes [38,39,40,41]. The combination of AX and other antioxidants, exerts an important protective effect reducing the free radical accumulation [42,43]. The primary natural source of AX (NAT-AX) is the unicellular microalgae *Haematococcus pluvialis*, which grows in fresh water all over the world and contains AX in complex with other carotenoids present in small quantities [41]. Alternative sources of AX are the synthetic form derived from petrochemicals (SYN-AX) and AX produced from genetically manipulated yeasts (PH-AX). Among these three sources, NAT-AX shows greater biological and antioxidant activities [44,45]. AX works as an antioxidant and anti-inflammatory therapeutic agent for cardiovascular diseases [42,46,47]. Indeed, it appears to protect cell membranes against lipid peroxidation, oxidative damage due to cholesterol, and low-density lipoproteins effects [38,48].

The combined effect of MK and AX in dyslipidemic subjects should be investigated. Accordingly, the purpose of the present study is to verify the mid to long-term physiological effects of a supplementation of MK and AX combined with a low energy/fat diet in a group of subjects with mild myopathies. We expect a significant improvement in biochemical parameters (e.g., lipid and glucose profiles and endothelial functions).

## 2. Materials and Methods

### 2.1. Participants

Eighty subjects (44 women, M age = 52.25, SD = 14.68, M BMI = 31.91, SD = 6.35) took part in this prospective observational study. Subjects were selected from the clinical database of the Human Physiology Section and Human Dietetics Service of the Department of Experimental Medicine of the University of Campania “Luigi Vanvitelli”, where all patients are registered. Participants were referred to our department by their general practitioners for the dietary management of dyslipidemia. Subjects were enrolled if they satisfied the following criteria: (1) they had interrupted treatment, according to their physician, with statins (no longer than 1 month prior to the study) because of issues with tolerability; (2) they had not or had not recently assumed (in the previous two weeks leading up to the study) a combined hypercholesterolemic treatment based on MK and AX as alternative treatment; (3) they had clinical symptoms consistent with myopathy (e.g., muscle weakness, myalgia and muscle cramps).

For each subject, a pathological and physiological anamnesis was carried out. Participants were selected according to the following inclusion/exclusion criteria: (i) age ≥ 18 years; (ii) diagnosis of dyslipidemia, defined as the presence of one or more disorders such as hypercholesterolemia, with total cholesterol (TC) levels ≥ 200 mg/dL, high-density-lipoprotein cholesterol (HDL-C) < 40 mg/dL for males and < 50 mg/dL for females, LDL-cholesterol (LDL-C) > 130 mg/dL, triglycerides (TG) ≥ 150 mg/dL; (iii) CPK levels (≥190 U/L); (iv) not pregnant or breastfeeding; (v) no proven intolerances to any component of the nutraceutical compound; (vi) no presence of liver or renal or CVD diseases; (vii) absence of type 1 or 2 diabetes mellitus; (viii) no chronic treatment with corticosteroid drugs.

### 2.2. Procedure

Participants enrolled in the study were selected because they were intolerant to statins, reported muscle weakness and myalgia, and showed increased CPK levels as reported by their physician. They were assigned to either the experimental (EG, *n* = 40, 24 women, M age = 53.05, SD = 12.82, M BMI = 30.56, SD = 6.03) or control group (CG, *n* = 40, 20 women, M age = 51.45, SD = 16.46, M BMI = 33.26, SD = 6.45) according to ongoing treatment of dyslipidemia. Participants included in the EG assumed an orally administered daily nutraceutical supplement containing 5 mg of MK + 0.1 mg of AX, as indicated by their physician. The AX that was used was NAT-AX derived from *Haematococcus pluvialis* powder at 5% of AX, containing 100% “S” enantiomer 3S, 3’s. Participants included in the CG had the same clinical features but were not taking daily supplementation. All participants (EG + CG) were prescribed individually tailored low-energy/fat diets depending on the basal metabolism. Energy, micronutrient, and macronutrient requirements were determined considering individual anthropometric characteristics in order to ensure that individual specifics nutritional needs were met. In general, each diet included 1200–1500 Kcal/day divided into 5–6 meals a day and consisting of 55–60% carbohydrates, 20–25% proteins, and 15–20% lipids; with trans-fats limited to <7% of total fats, saturated fats <1/3 of total fat intake, cholesterol intake <300 mg/day; 4–5 portions/day of fruits and vegetables. For all subjects, no indication of variations in the physical activity levels has been prescribed. To fulfil our aim, age, body mass index (BMI) and biochemical parameters, i.e., glycemia, TC, HDL-C, LDL-C, TG, CRP, and CPK, were collected in both groups at baseline (*T*_0_), after 12 weeks (*T*_1_) and 24 weeks (*T*_2_). Monthly dietary plans were modified according to any emerging individual needs and bioimpedance results, where necessary. Adherence to the program was checked monthly with follow-up examinations, and there were no drop-outs.

### 2.3. Ethics Statement

All participants gave prior written informed consent, which was submitted to the ethics committee of the University of Campania “Luigi Vanvitelli” with Protocol Number 126265/2022-UNA2CLE-0126265 ID2353955 and carried out according to the 1964 Declaration of Helsinki in order to participate in this study.

### 2.4. Statistical Analyses

To verify between-group equivalence on demographic, anthropometric, and biochemical variables at baseline, EG and CP were compared by means of two-way chi-square test (χ^2^) for nominal variables and univariate Analysis of Variance (ANOVA) for continuous variables. For descriptive purposes, means ± standard deviations (BMI and biochemical parameters) were reported for each time. Then, a 2 × 3 mixed factorial ANOVA was performed to weight any principal and/or interaction effects between time and treatment. Specifically, eight models (outcome variables: BMI, glycemia, TC, HDL-C, LDL-C, TG, CRP, and CPK) were constructed, with treatment (EG vs. CG) that was the between-subject factor and time (T0 vs. T1 vs. T2) the within-subject factor. The Greenhouse–Geisser correction was applied to the degrees of freedom if data did not meet the assumption of sphericity according to Mauchly’s *W*. In the case of a principal effect of time or time × treatment interaction effect, post-hoc analyses were performed by applying Bonferroni’s correction for multiple comparisons (*p*_bonf_). Effect size was estimated by partial eta-squared ($\eta_{p}^{2}$). A *p*-value lower than 0.05 was considered statistically significant. All statistical analyses were performed by IBM SPSS Statistics for Windows, version 26 (IBM Corp., Armonk, NY, USA) and JASP package.

## 3. Results

Univariate outliers (i.e., *z*-scores = |3|) were removed. If needed, square root transformation (√X_i_) was performed to normalize variables in line with skewness and kurtosis parameters (i.e., < |1|) [49,50]. For multivariate diagnostics of outliers, the Mahalanobis’ distance ($D_{i}^{2}$) was calculated and Mardia’s coefficient was assessed. Between-group equivalence was confirmed according univariate ANOVAs (BMI: *F*(1, 79) = 3.73, *p* = 0.59; Glycemia: *F*(1, 79) = 2.07, *p* = 0.154; Serum total cholesterol levels: *F*(1, 79) = 0.402, *p* = 0.528; LDL-cholesterol levels: *F*(1, 79) = 0.179, *p* = 0.673; HDL-cholesterol: *F*(1, 79) = 1.77, *p* = 0.187; Triglycerides: *F*(1, 79) = 0.48, *p* = 0.828; CRP: *F*(1, 79) = 0.397, *p* = 0.531; CPK: *F*(1, 79) = 3.27, *p* = 0.062). Descriptive statistics for each time were reported in Table 1.

### 3.1. Mixed Factorial ANOVA

#### 3.1.1. Body Mass Index

**Figure 1:**
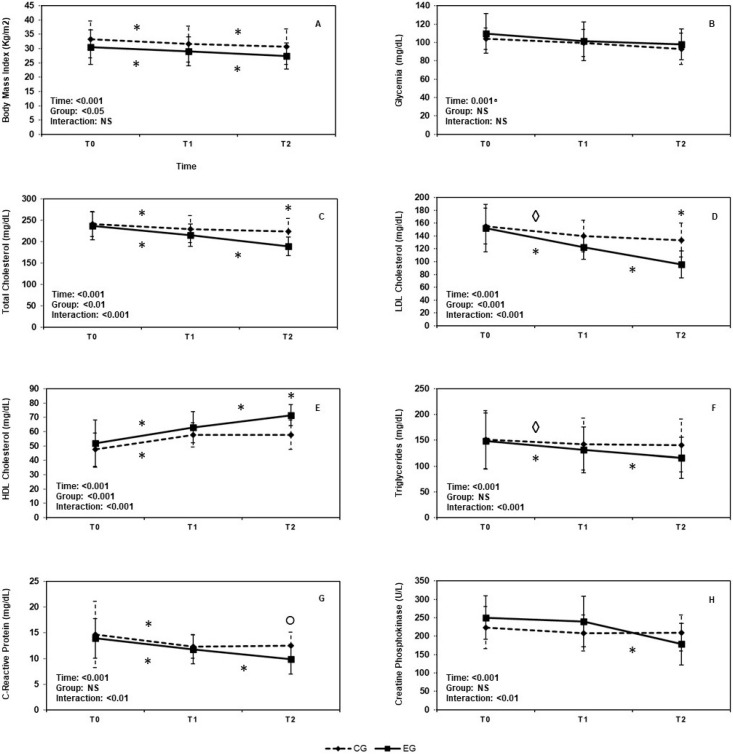
Line plots representing the results from mixed factorial ANOVAs on biochemical parameters in experimental group (EG) and control group (CG): (**A**) BMI, (**B**) glycemia, (**C**) total cholesterol, (**D**) LDL-cholesterol, (**E**) HDL-cholesterol, (**F**) triglycerides, (**G**) C-reactive protein, and (**H**) creatine phosphokinase. *Note*. * *p* < 0.001, ◊ *p* < 0.01, ○ *p* < 0.05; ^a^ T0–T2 for both groups, *p* < 0.05.

We found a main effect of time (*F*(1, 91) = 184.85, *p* < 0.001, $\eta_{p}^{2}$ = 0.71) and treatment (*F*(1, 78) = 4.83, *p* = 0.03, $\eta_{p}^{2}$ = 0.06). Both groups exhibited a significant weight decrease over time (all pairwise comparisons *p*_bonf_ < 0.001); however, if data were averaged over the time levels, participants within EG showed a lower BMI than those in CG (mean diff: −2.83, SE = 1.29, *p*_bonf_ = 0.03). No interaction time × treatment (*F*(1, 91) = 3.13, *p* = 0.07) emerged (see Figure 1A).

#### 3.1.2. Glycemia

A main effect of time (*F*(1, 86) = 11.63, *p* = 0.001, $\eta_{p}^{2}$ = 0.13) was observed, with glycemia levels decreasing over time in both groups. Conversely, no main effect of group (*F*(1, 78) = 2.20, *p* = 0.14) or time × treatment interaction effect (*F*(1, 86) = 0.36, *p* = 0.57) were shown (see Figure 1B).

#### 3.1.3. Serum Total Cholesterol Levels

We found a main effect of time (*F*(1, 111) = 170.60, *p* < 0.001, $\eta_{p}^{2}$ = 0.69), treatment (*F*(1, 78) = 8.13, *p* = 0.006, $\eta_{p}^{2}$ = 0.09), and a significant interaction between time and treatment (*F*(1, 111) = 37.46, *p* < 0.001, $\eta_{p}^{2}$ = 0.32). Specifically, both groups exhibited a significant decrease in serum total cholesterol levels over time (all pairwise comparisons *p*_bonf_ < 0.001); however, if data were averaged over the time levels, participants within EG showed lower serum total cholesterol levels overtime than those in CG (mean diff: −17.63, SE = 6.18, *p* = 0.006). Finally, taking the levels of the time variable separately, EG showed a significant decrease in serum total cholesterol levels only from T1 to T2 (T0, mean diff: −0.05, SE = 5.10, *p*_bonf_ = 0.99; T1, mean diff: −13.90, SE = 5.10, *p*_bonf_ < 0.11; T2, mean diff: −34.55, SE = 5.10, *p*_bonf_ < 0.001) (Figure 1C).

#### 3.1.4. LDL-Cholesterol Levels

Main effects of time (*F*(1, 116) = 94.80, *p* < 0.001, $\eta_{p}^{2}$ = 0.55), treatment (*F*(1, 78) = 15.70, *p* < 0.001, $\eta_{p}^{2}$ = 0.17), and a significant time × treatment interaction effect (*F*(1, 116) = 18.86, *p* < 0.001, $\eta_{p}^{2}$ = 0.19) were found. Both groups exhibited a significant decrease in LDL over time (all pairwise comparisons *p*_bonf_ < 0.001). If data were averaged over the time levels, participants within EG showed lower LDL than those in CG (mean diff: −19.62, SE = 4.95, *p* < 0.001); taking the levels of the time variable separately, EG showed a significant decrease in LDL from T1 to T2 (T0, mean diff: −3.10, SE = 5.95, *p*_bonf_ = 0.99; T1, mean diff: −17.70, SE = 5.95, *p*_bonf_ = 0.05; T2, mean diff: −38.05, SE = 5.95, *p*_bonf_ < 0.001) (Figure 1D).

#### 3.1.5. HDL-Cholesterol Levels

Main effects of time (*F*(1, 111) = 139.03, *p* < 0.001, $\eta_{p}^{2}$ = 0.64), treatment (*F*(1, 78) = 11.77, *p* = 0.001, $\eta_{p}^{2}$ = 0.13), and a significant time × treatment interaction effect (*F*(1, 111) = 15.29, *p* < 0.001, $\eta_{p}^{2}$ = 0.16) were shown. Both groups exhibited a significant increase in HDL over time (all pairwise comparisons *p*_bonf_ < 0.001); however, if data were averaged over the time levels, participants within EG showed higher HDL than control participants (mean diff: 7.78, SE = 2.27, *p* = 0.001). By taking the levels of the time variable separately, EG showed a significant increase in HDL from T1 to T2 (T0, mean diff: 4.25, SE = 2.51, *p*_bonf_ = 0.99; T1, mean diff: 5.40, SE = 2.51, *p*_bonf_ = 0.50; T2, mean diff: 13.70, SE = 2.51, *p*_bonf_ < 0.001) (Figure 1E).

#### 3.1.6. Triglycerides Levels

We detected a significant effect of time (*F*(1, 102) = 92.68, *p* < 0.001, $\eta_{p}^{2}$ = 0.54; all pairwise comparisons, *p*_bonf_ < 0.001) and time × treatment interaction effect (*F*(1, 111) = 22.40, *p* < 0.001, $\eta_{p}^{2}$ = 0.22) but no effect of treatment (*F*(1, 78) = 1.33, *p* = 0.25) (Figure 1F).

#### 3.1.7. C-Reactive Protein, CRP

A main effect of time (*F*(1, 93) = 51.54, *p* < 0.001, $\eta_{p}^{2}$ = 0.40) and a significant time × treatment interaction effect (*F*(1, 93) = 6.23, *p* = 0.003, $\eta_{p}^{2}$ = 0.07) were found. Both groups exhibited a significant decrease in CPR over time (all pairwise comparisons *p*_bonf_ < 0.001). On average, EG did not differ significantly in CPR levels as compared to CG (mean diff: −1.32, SE = 0.76, *p* = 0.08); however, by taking the levels of the time variable separately a difference emerged, with EG showing a significant decrease in CPR from T1 to T2 (T0, mean diff: −0.75, SE = 0.84, *p*_bonf_ = 0.99; T1, mean diff: −0.61, SE = 0.84, *p*_bonf_ = 0.99; T2, mean diff: −2.61, SE = 0.84, *p*_bonf_ = 0.04) (Figure 1G).

#### 3.1.8. Creatin Phosphokinase, CPK

We found a main effect of time (*F*(1, 121) = 27.84, *p* < 0.001, $\eta_{p}^{2}$ = 0.26) and a significant time × treatment interaction effect (*F*(1, 121) = 16.79, *p* < 0.01, $\eta_{p}^{2}$ = 0.18); no main effect of treatment (*F*(1, 78) = 0.72, *p* = 0.40) was shown. Both groups exhibited a significant decrease in CPK over time (all pairwise comparisons *p*_bonf_ < 0.001). Furthermore, the interaction effect was justified by the lack of a significant difference between T1 and T2 in the CG (mean diff: −0.60, SE = 8.41, *p* = 0.99), while a significant decrease between T1 and T2 was found in EG (mean diff: −61.15, SE = 8.41, *p* < 0.001) (Figure 1H).

## 4. Discussion

Our results showed that subjects receiving the combined treatment of a low-energy/fat diet and daily nutraceutical supplementation (5 mg of MK + 0.1 mg of AX) achieved significant improvement in many of the investigated biochemical parameters. BMI, TC, LDL-C, HDL-C, CRP and CPK values significant improved after 24 weeks. In particular, we observed a beneficial effect of combined treatment as early as 12 weeks and further significant improvement after 24 weeks. Specifically, after a 12-week treatment, subjects belonging to the EG showed a significant decrease in TC (EG −9.04% vs. CG −4.96%) and LDL-C (EG −19.79% vs. CG −10%) levels. Furthermore, while EG and CG did not differ in terms of HDL-C improvement after 12 weeks (EG +22.05% vs. CG +21,6%), we observed a significant increase in HDL-C in the experimental group after 24 weeks (EG +13.31% vs. CG +0.17%). Thus, after 24 weeks of treatment, EG subjects showed a significant increase in HDL-C compared to CG (EG 38.30% vs. CG 21.81%). Still, after 24 weeks, EG showed a general improvement in TC (EG −20.21% vs. CG −7.37%), LDL-C (EG −37.20% vs. CG −13.96%), TG (EG −22.16% vs. CG −7.45%) values. Such results could be a consequence of the nutraceutical treatment. As suggested [39], the combined effect of nutraceuticals containing both MK and AX, with a low energy/fat dietary regimen, would be more effective in improving the lipid profile than the low-energy/fat diet alone. These outcomes are similar to those achievable via low doses of statins [51,52]. It has been highlighted that MK improves lipid profile inhibiting HMG-CoA reductase in a statin-like action [51]. In particular, the dosage considered in our study (5 mg of MK + 0.1 mg of AX) appears effective and well-tolerated. Significant reductions of CPK values in the EG, mainly after 24 weeks of treatment, support the improvement of myopathy-symptoms (EG −28.76% vs. CG −6.50%). 

A chronic low-grade inflammation is a common feature of obesity [53]. It has been suggested that mitochondrial dysfunction in the liver, adipose tissue and skeletal muscle is related to this inflammation which causes reduced fatty acid oxidation, increased accumulation of reactive oxygen species (ROS), and impaired mitochondrial biogenesis [53,54,55,56]. The mechanism underling this dysfunction could be attributed to a decreased level of AMP-activated protein kinase (AMPK) activity [53]. Indeed, AMPK regulates cellular energy homeostasis and mitochondrial biogenesis stimulating mitochondrial fatty acid oxidation and reducing fatty acid synthesis [57,58,59,60]. AMPK activation promotes fatty acid oxidation stimulating PPARα [61]. In fact, PPARα stimulates the transcription of genes that promote mitochondrial biogenesis and mitochondrial oxidation of fatty acids and ketogenesis, increases apolipoproteins A-I and A-II and increases HDL cholesterol levels [39]. PPARα also stimulates hepatic production of Fibroblast Growth Factor 21 (FGF21) [62], which increases adiponectin production by adipocytes that, in turn, stimulates AMPK activity in liver and other tissues [62,63]. AMPK also stimulates PPARγ coactivator-1a (PGC-1a) activity, which acts as a coactivator for PPARα [61,64,65]. 

Considering our results, several factors might be responsible for the synergistic effects on lipid metabolism. On the one hand, MK, which acts similarly to statins, can be responsible for the hypolipidemic effects observed by reducing cholesterol biosynthesis [4,25,27]; on the other hand, AX stimulates PPARγ activity, which improves the lipid profile, reduces hepatic lipid accumulation and increases adiponectin and HDL-C levels. In fact, AX has antioxidant and anti-inflammatory effects modulating inflammatory signaling pathways such as NF-kB (nuclear factor kappa-light-chain-enhancer of activated B cells) and PPARγ, and downregulating the expression of inflammatory cytokines [66]. It has also been demonstrated that AX has a higher antioxidant activity than vitamin E and vitamin C, enhancing stress resistance [37,41,66]. On biological membranes, AX exerts antioxidant effects, inhibiting mitochondrial lipid peroxidation and reducing oxidative stress [47,67]. Furthermore, by acting on the gene expression of lipid metabolism of hepatocytes and as an agonist of PPARα, AX improves the lipid profile and reduces the accumulation of liver lipids [38,48]. Referring to adiponectin, it enhances mitochondrial biogenesis, modulates glucose and lipid metabolism in skeletal muscle, controls energy homeostasis, and modulates inflammation activating AMPK [68,69,70,71,72]. Furthermore, it reduces serum TG levels by stimulating Very-Low-Density Lipoprotein (VLDL) catabolism increasing HDL-C in AX-treated individuals [67]. The mechanism proposed is that adiponectin stimulates Apolipoprotein A-1-mediated cholesterol efflux from macrophages through ATP-binding cassette transporter A1-dependent pathway by activating PPARγ [73]. All these factors may have contributed to the modulation of low-grade inflammation characteristic of obesity by giving the additional benefits derived from combining with an antioxidant. Marazzi and colleagues [74] evaluated the effectiveness and safety of a nutraceutical combination with RYR and antioxidants after 3, 6 and 12 months of treatment without any dietary intervention. After 12 months they showed a significant reduction in TC (−20%) and LDL-C (−31%), but no change in HDL-C. In contrast, our results showed a reduction in TC, LDL-C and TG, but also an increase in HDL-C after 24 weeks. This may be due to the synergistic effect of dietary intervention and nutraceutical supplementation.

However, previous findings on the effects of MK and AX are mixed. Ruscica and colleagues [52] found no significant improvement in CPK and CRP after 8 weeks of nutraceutical supplements including MK and AX. Conversely, other studies have shown a decrease in CRP after only AX treatment [66,75,76]. In our study, we observed an improvement in both CPK and CRP, highlighting an important anti-inflammatory effect of this combination therapy, especially from 12 to 24 weeks. Weight loss is usually associated with a decrease in chronic low-grade inflammation typically of obesity [53,77,78,79,80]. However, although both groups had a similar variation in weight loss after 24 weeks (EG −10.41% vs. CG −7.91%), the EG group showed a significant decrease in CRP (EG −29.12% vs. CG −14.94%). These results support the anti-inflammatory synergistic effect of nutraceutical supplementation combined with diet. 

It is important to stress the large variability in the amount of MK and AX in the different available commercial preparations [30]. Therefore, there is a need to promote the careful evaluation of dietary supplements to better characterize their safety profile and identify the best nutraceutical combination with a lower side effect profile [3,25].

In summary, our findings confirm the efficacy of the combined use of MK and AX in the treatment of dyslipidemia in concert with a low-energy/fat diet. This could be explained by a synergistic effect of the components of the nutraceutical supplementation and dietary intervention that reduce dyslipidemia and the low-grade inflammation characteristic of obesity. The safety and tolerability of this nutraceutical combination appears to be encouraging; in particular, the dosage used in our study seems to be adequate for achieving beneficial effects, also in terms of reducing side effects such as myopathies.

This study presents some limitations that may impact the generalizability of the results. First, this is an observational study based on evidence from ambulatory clinical practice. This made not possible to manipulate the experimental variables (i.e., different amount of MK and AX, comparisons with other lipid-lowering and/or placebo treatment groups). Second, changes in body composition should be confirmed using DEXA for assessing changes in visceral adipose tissue. Additionally, physical activity levels were not evaluated. It could be interesting to explore the combined effect of diet interventions, nutraceutical supplements and levels of physical activity. It has a strong influence on the lipid profile, affecting both obesity and dyslipidemia. For these reasons, other studies are required.

## 5. Conclusions

Our results highlight that the combined effects of low-energy/fat diet and daily nutraceutical supplementation (5 mg of monacolin K + 0.1 mg of astaxanthin) could be considered as an effective sparkling approach to improving the lipid profile. This treatment might be an alternative therapeutic strategy, in concert with a hypocaloric diet, for patients who do not tolerate statins or in the case of mild dyslipidemia. Our results emphasize and underscore that it is necessary to rely on appropriate nutraceutical supplementation in conjunction with an appropriate diet, when applicable, before using drugs that could have non-negligible side effects. Future research could clarify the long-term effects of nutraceutical therapy.

## Figures and Tables

**Table 1:** Descriptive characteristics for each time for EG and CG.

	T0		T1		T2	
	CG	EG	CG	EG	CG	EG
BMI, mean (SD)	33.26 (6.45)	30.56 (6.03)	31.58 (6.32)	29.03 (5.1)	30.63 (6.21)	27.38 (4.53)
Glycemia mg/dL, mean (SD)	104.18 (11.59)	109.75 (21.58)	99.45 (14.57)	101.25 (21.26)	93 (17.04)	97,95 (16.74)
TC mg/dL, mean (SD)	241.50 (29.8)	237.05 (32.92)	229.50 (32,01)	215.60 (26.18)	223.7 (30.76)	189.15 (21.66)
LDL-C mg/dL, mean (SD)	155.40 (27.98)	152.30 (36.86)	139.85 (24.61)	122.15 (18.48)	133.7 (26.49)	95.65 (21.37)
HDL-C mg/dL, mean (SD)	47.45 (11.56)	51.70 (16.57)	57.70 (8.43)	63.10 (10.81)	57.80 (10.31)	71.50 (7.36)
TG mg/dL, mean (SD)	151.65 (56.21)	148.95 (54.38)	142.45 (50.49)	131,60 (44.33)	140.35 (51.3)	115.95 (39.98)
CRP mg/L, mean (SD)	14.66 (6.47)	13.91 (3.86)	12.35 (2.33)	11.74 (2.82)	12.47 (2.66)	9.86 (2.89)
CPK U/L, mean (SD)	223.49 (56.9)	250.31 (59.05)	208.37 (48.83)	239.47 (68.6)	208.97 (48.63)	178.32 (56.62)

BMI: body mass index; TC: serum cholesterol; LDL: low-density lipoprotein; HDL: high-density lipoprotein; TG: triglycerides; CRP: C-reactive protein; CPK: creatin phosphokinase; T0: baseline; T1: 12 weeks; T2: 24 weeks; CG: control group; EG: experimental group.

## Data Availability

Not applicable.

## Sample Availability

Samples of the compounds are available from the authors.
